# Standard Assays for the Study of Autophagy in the Ex Vivo Retina

**DOI:** 10.3390/cells6040037

**Published:** 2017-10-22

**Authors:** Raquel Gómez-Sintes, Beatriz Villarejo-Zori, Ana Serrano-Puebla, Lorena Esteban-Martínez, Elena Sierra-Filardi, Ignacio Ramírez-Pardo, Natalia Rodríguez-Muela, Patricia Boya

**Affiliations:** Departament of Cellular and Molecular Biology, Centro de Investigaciones Biológicas, CSIC, 28040 Madrid, Spain; bvillarejo@cib.csic.es (B.V.-Z.); anaserrano@cib.csic.es (A.S.-P.); lestebanm@cnic.es (L.E.-M.); esierra@cib.csic.es (E.S.-F.); naxo_nx3@hotmail.com (I.R.-P.); natalia2810@hotmail.com (N.R.-M.)

**Keywords:** autophagy, retina, organotypic cultures, lysosomes, mitophagy

## Abstract

Autophagy is a catabolic pathway that mediates the degradation and recycling of intracellular components, and is a key player in a variety of physiological processes in cells and tissues. Recent studies of autophagy in the eye suggest that this pathway is fundamental for the preservation of retinal homeostasis. Given its accessible location outside the brain, the retina is an ideal organ in which to study the central nervous system and a wide range of neuronal processes, from development to neurodegeneration. Here we review several methods used to assess autophagy in the retina in both physiological and pathological conditions.

## 1. Introduction to Autophagy

Autophagy is a process which primarily occurs in response to stress, enabling the recycling of proteins, lipids, and whole organelles to generate the building blocks required to sustain cellular homeostasis [1,2]. Autophagy is also implicated in cellular quality control, particularly in neurons, in which altered proteins and damaged organelles cannot be redistributed to daughter cells through cell division [3]. This pathway begins with the formation of the autophagosome. This double-membrane organelle engulfs cell components destined for degradation, and delivers them to the lysosome for elimination. The autophagy process is regulated by the *Atg* family of proteins, which is highly conserved across eukaryote kingdom. Given its important cytoprotective role in response to stress, dysregulation of autophagy results in many pathophysiological alterations, and is implicated in a range of pathologies, from cancer to neurodegeneration.

Only recently has research focused on the role of autophagy in the visual system. The eye, and in particular the retina, is exposed to a variety of environmental insults and stressors, including gene mutations and age-related changes that lead to functional impairment [4]. The development of new therapeutic strategies for retinal diseases requires a better understanding of the role of autophagy in retinal homeostasis.

## 2. The Retina, an Ideal Model for the Study of Autophagy in the Central Nervous System

**Figure 1:**
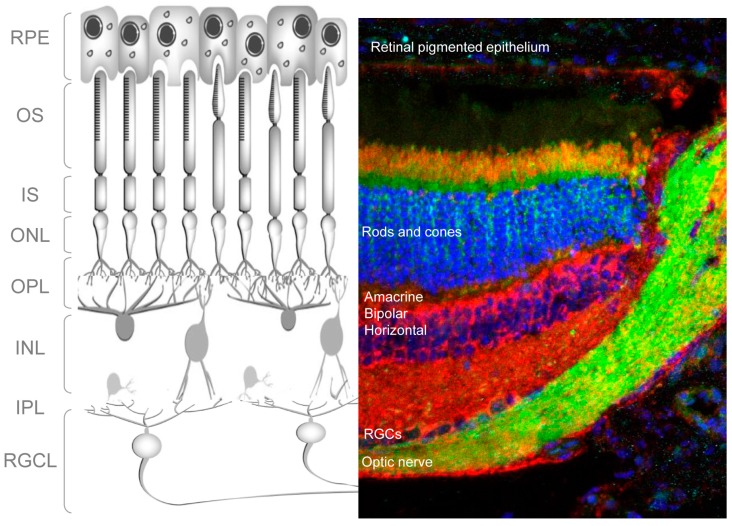
Morphology of the adult mouse retina. (**Left**) Schematic depicting the different cell types in the adult mouse retina; (**right**) retinal section from an adult GFP-LC3 mouse (green) stained with TOMM20 (SantaCruz Biotechnology sc-11415, in red) to assess mitochondria and DAPI (in blue) to stain nuclei.

The retina is a light-sensitive tissue in the vertebrate eye that detects and processes visual images. It does this by sensing light and creating impulses, which are then carried to the brain via the optic nerve. Structurally, the retina consists of multiple cell types arranged in layers (Figure 1); specifically, three layers of neuronal cell bodies and two layers of synapses. Light-sensitive photoreceptors (rods and cones) make up the outer nuclear layer (ONL) (Figure 1). In the outer plexiform layer, these cells form connections with amacrine and bipolar cells whose nuclei, in turn, lie in the inner nuclear layer (INL). In the inner plexiform layer amacrine and bipolar cells synapse with the retinal ganglion cells (RGCs). These are the only projecting neurons of the retina, and their axons form the optic nerve, which connects the retina to the brain (Figure 1). Other important cells of the retina are the retinal glia, or Müller cells, located around the RGCL and the retinal-pigmented epithelial (RPE) cells, which lie immediately outside the neuroretina, in close contact with the photoreceptors. RPE cells provide trophic support to photoreceptors and mediate the recycling of photoreceptor outer segments.

The retina has several unique features that make it an ideal window into the CNS, enabling the study of a range of neuronal processes, from development to neurodegeneration [5]. Crucially, its location outside of the brain makes it the most accessible part of the CNS. Moreover, the retina can be cultivated organotypically under semi-physiological conditions, in which cell-to-cell and cell-to-matrix communication is maintained.

Autophagy is essential for proper retinal development and vision [5,6], and dysregulation of this process is implicated in many retinal pathologies (for a review of studies of autophagy in the visual system, see [7]). As with any emerging field of research, the rate of progress in autophagy research is dependent on the availability of robust tools that are applicable to different biological conditions and experimental settings. Since the “molecular era” the tools used in autophagy have been continuously improved and refined. Classical ultrastructural analytical methods have gradually been replaced by new tools that are easier, faster, and require less expertise (for a comprehensive review of tools used to monitor autophagy in different models, see [8]).

Here, we describe several methods that we have developed to study autophagy in the visual system, with a specific focus on the retina. We begin with a short description of the isolation and ex vivo culture of the retina. In addition, we discuss the range of assays that we routinely use to assess as the expression of *Atg* genes at the transcriptional level, standard assays used to monitor LC3 lipidation that is used to monitor autophagy at the steady state level. Autophagic flux using lysosomal inhibitors can be determined by Western blot in ex vivo (the retina is cultured) and in vivo (where proteases and inhibitors are injected in the animal) samples. Finally, immunofluorescence and flow cytometry approaches are used to assess autophagy proteins and substrates.

## 3. Ex Vivo Retinal Culture

**Figure 2:**
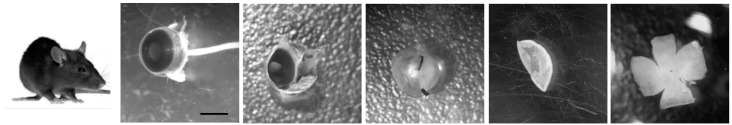
Isolation of adult mouse retina. After dissection, the retina is flattened by creating four incisions from the periphery to the centre, as shown in the last panel. This allows better adherence to a nitrocelullose membrane for easier handling. Scale bar: 2 mm.

The cultured retina remains viable for long periods in defined medium and is, thus, particularly useful for autophagy research. For example, altering the composition of the culture conditions allows manipulation and assessment of the autophagy-dependent response to nutrient and growth factor availability. An additional advantage of serum-free media is that batch-to-batch differences in serum composition are reduced. In our laboratory, we use both embryonic and adult mouse retinas, with slight variations in culture conditions depending on the age of the retina and the required duration of incubation. After sacrifice, eyes are enucleated and placed in cold PBS. Under a binocular dissecting microscope, a pair of fine tweezers is used to separate the neural retina from the other components of the eye; first from the sclera and pigment epithelium, then from the ora serrata and, at last, from the lens. Finally, after making a series of incisions from the edge towards the centre, the retina is flat-mounted, as shown in Figure 2. Due to the reduced size of the embryonic retina it can be directly mounted into the nitrocellulose filter without making incisions.

We cultured embryonic mouse retinas for up to 6 h in DMEM/F12 medium containing N2 supplement (Gibco 17502-048). The process of neurogenesis is unaltered in these culture conditions, no increase in cell death is observed with respect to normal in vivo retinal development and under these conditions, insulin acts as the main pro-survival factor [9]. Embryonic avian retinas have also proved highly useful for autophagy research, like the mouse retina, as the manner in which developmental processes, such as cell death, phagocytosis, and neurogenesis, occur in cultured avian retinas closely resembles the in vivo situation [6,10].

Postnatal and adult rodent retinas are cultured in membrane inserts (e.g., Millicell by Millipore, Billerica, MA, USA), made from mixed cellulose esters, that float in the culture medium. On those inserts, the retina is placed with the photoreceptors facing down in order to mimic physiological nutrient delivery to the retina. For short experiments (up to 24 h), retinas are cultured in Millicell inserts in insulin-supplemented DMEM. For longer experiments, the adult retina is cultured in R16 medium which has a more complex composition with many other supplements such as tocopherol, ascorbic acid, and retinol [11].

It is important to note that in these organotypic cultures, the optic nerve is severed, thus subjecting the retina and retinal ganglion cells to additional stress. We have observed an increase in the levels of cell death during the first 24 h of culture in the photoreceptor layer and degeneration of RGCs with longer culture times (over three days). Thus, when assessing the neuroprotective effects of compounds in models of retinal diseases, it is important to perform the same treatments in non-diseased or wild-type retinas to compare with tissue culture associated cell death.

Autophagy is usually induced by incubating retinas in amino acid-free medium (EBBS) for up to 6 h, or with mTOR inhibitors such as rapamycin, Torin, or AZD8055 (Table 1). Ex vivo cultures allow the administration of pharmacological treatments such as protease inhibitors, which block lysosomal degradation to enable the analysis of autophagic flux. After culture for the desired duration, the retina can be used for subsequent biochemical and cellular analyses, as described below.

## 4. Transcriptomic Analysis in Retinal Explants

While autophagy is mainly regulated at the postranscriptional level, high rates of autophagosome formation require increased mRNA expression of *Atg* genes [12]. One example of this phenomenon is the increase in *Atg*5 mRNA expression observed following optic nerve axotomy in mice [13]. Conversely, decreased mRNA expression of autophagy regulators, such as *Atg*7 or *Beclin1*, in the mouse retina have been associated with increasing age [14].

mRNA expression of autophagy genes can be determined in a single adult mouse retina. By contrast, due to the limited amount of tissue in embryonic retinas, pools of two retinas can be used. This approach is useful to study autophagy-deficient animals, which need to be genotyped on the same day as the experiment. An alternative approach to reduce animal-to-animal variability is to create pools of several retinas (e.g., for RNA sequencing) [15]. All these assays, including transcriptomic analyses, can be performed using either freshly dissected tissue or following ex vivo explant culture.

## 5. Determination of Autophagic Flux by Western Blot in Retinal Explants

Autophagosome formation is a key feature of autophagy, and can be directly assessed by monitoring levels of the autophagosomal binding protein MAP-LC3 (microtubule-associated protein LC3) [16]. Autophagosome formation involves *Atg*7-mediated lipidation of LC3 (the mammalian ortholog of *Atg*8), by covalent attachment of phosphatidyl ethanolamine on the autophagosomal membrane. This lipidation process can be detected by Western blot: conjugated LC3 (LC3-II) progresses faster through the gel than non-conjugated LC3, and is visualized as a lower molecular-weight band [17], allowing determination of the free and autophagosome-bound forms of LC3. Additionally, other *Atg*8-GABARAP family members or p62 accumulation serve to monitor autophagic flux.

While LC3 levels provide an indication of autophagosome number at a given moment, they do not provide a direct read-out of autophagy activity per se. For example, autophagosome number can also be increased by autophagy blockade at later stages of the process (e.g., by blocking degradation using lysosomal inhibitors) [18,19]. Autophagic flux encompasses the entire dynamic process of autophagy and is, therefore, a more reliable indicator of autophagic activity [16]. This is determined by comparing autophagosome levels in control conditions with those observed in the final stages of the process (i.e., after lysosomal inactivation). The use of this method in vitro is well described, and it can also be applied to ex vivo samples: cells or retinal explants are incubated in the absence or presence of lysosomal inhibitors, and autophagosome levels subsequently determined by Western blot [16].

**Figure 3:**
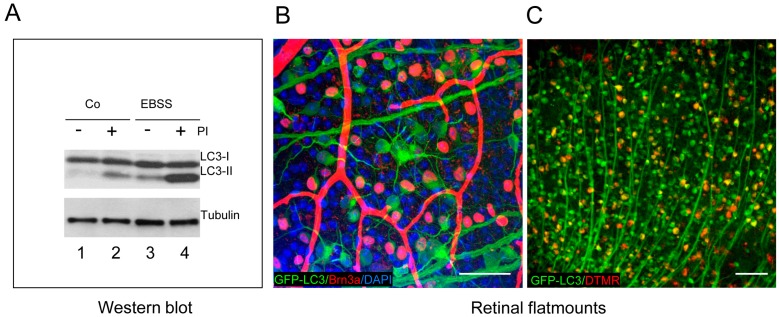
Assessment of autophagic flux by western blot and immunostaining of retinal flatmounts. (**A**) E13.5 embryonic retinas are incubated with or without EBSS to induce autophagy and incubated in the presence (+) or absence (−) of PI (ammonium chloride and leupeptin). Note the differential electrophoretic mobility of the non-lipidated (LC3-I) and lipidated (LC3-II) LC3 bands; (**B**) assessment of autophagy in GFP-LC3 retinal flatmounts (in green) from adult mouse stained with the RGC marker Brn3a (Millipore MAB1585, in red). Nuclei are stained with DAPI (in blue). Note the non-specific binding of the secondary antibody (in red) to the retinal vessels; and (**C**) retrograde labelling of RGCs (in red) in the GFP-LC3 mouse (adult, in green) by injection of the RGC-specific dye DTMR (Dextran tetramethylrhodamine, Molecular Probes, in red). Scale bars 50 μm.

In our laboratory, we have assessed autophagic flux in mouse retinal explants after amino acid starvation. Embryonic neuroretinas at E13.5 are isolated and cultured in EBSS for 6 h to induce amino acid starvation in the absence (−) or presence (+) of chloroquine or leupeptin, which block lysosomal degradation. Chloroquine acts by increasing lysosomal pH, whereas leupeptin is a protease inhibitor (PI) (Table 1). Figure 3A shows a classical experiment to assess autophagic flux by measuring LC3 lipidation, which allows assessment of basal autophagy (compare lines 1 and 2), as well as starvation-induced autophagic flux (compare lines 3 and 4). Embryonic mouse retinas display some degree of basal autophagy (Figure 3A), as demonstrated when lysosomal proteases are inhibited with leupeptin [15,20]. Similar findings are observed in the chick retina at several embryonic stages [6,10]. Thus, using Western blot, both basal and induced autophagic flux can be detected ex vivo in embryonic and adult retinal explants. Moreover, autophagic flux can be determined in the retina in vivo in adult mice following intraperitoneal injection of lysosomal inhibitors. This technique produces results comparable to those obtained in ex vivo retinal explants [20]. It is important to note that autophagy in the retina is influenced by the circadian rhythm which exhibits a bimodal pattern that correlates with shifts in the transduction proteins within the photoreceptor and by circadian ingestion of outer segments in the RPE [21]. To control for this phenomenon, it is crucial that all retinas for a given experiment are isolated at the same time of day. In conclusion, the retina, as a part of the central nervous system, is a very useful model to determine autophagy flux, as it can be assessed ex vivo and in vivo, as some lysosomal inhibitors are able to cross the blood–retinal barrier which, in some instances, is more permeable than the blood–brain barrier [20].

## 6. Immunofluorescence in Flatmounts and in Eye Cryosections

GFP-LC3 transgenic mice, which express the GFP-LC3 reporter in all cells of the body via a constitutive promoter, are often used for the evaluation of autophagy in tissue. Autophagy has been demonstrated in vivo in these mice, and is induced in response to nutrient starvation in most tissues (liver, heart, pancreas, muscle, and kidneys), but not in the brain [22].

We recently used GFP-LC3 mice to assess autophagy flux in retinal sections by immunofluorescence. In mice injected with lysosomal inhibitors to assess autophagic flux, food deprivation increased autophagosome number in most retinal layers [20]. These data have two important implications: first, food deprivation induces autophagic flux in the retina; and second, as stated above, protease and lysosomal inhibitors can cross the blood–retinal barrier, further underscoring another unique feature of the retina that makes it an ideal model for in vivo study of the role of autophagy in the central nervous system.

Retinal flatmounts provide a useful means of studying autophagy in retinal ganglion cells, which are distributed as a single layer of cells and are easily observable in confocal sections of multiple z-planes. Autophagosome formation in RGCs can be monitored in GFP-LC3 retinal flatmounts stained with Brn3a, an RGC-specific marker (Figure 3B), and is increased following axonal damage in a mouse model of RGC death [13]. Interestingly, an increase in the number of puncta is observed in retinal flatmounts following optic nerve axotomy in GFP-LC3 mice, with specific upregulation of autophagy observed in the retinal ganglion cells whose axons are sectioned (Figure 3B). While blood vessels often display non-specific labelling with many secondary antibodies, LC3 can be readily observed in RGC axons. RGC-specific autophagy can also be assessed by retrograde labelling of RGCs, through the injection of fluorescent dyes, such as DTMR, into the superior colliculus. After 24 h, the dye is transported from the axon to the soma of the RGCs, which are identified as double-labelled cells (Figure 3C). This autophagy increase is a prosurvival response as rapamycin reduces cell RGC death in vivo after axonal damage [13]. However, under conditions of lysosomal damage, increasing autophagy seems to be detrimental, as evidenced by enhanced cell death in rd10 retinal explants treated with a calcium ionophore and the in vivo treatment of the rd10 mice with rapamycin and trehalose [23]. Interestingly both cell death and LC3 levels can be assessed in the same cell, by using double immunofluoresce with activated caspase-3 and LC3 antibodies.

**Figure 4:**
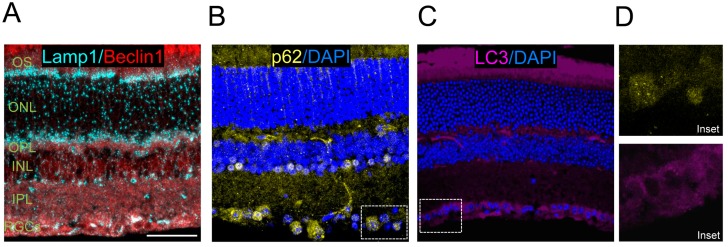
Immunolabelling of autophagy proteins in eye cryosections from wild type mouse. (**A**) Lysosomes are immunolabeled using antibodies against Lamp1 (1D4B Developmental Sudies Hibridoma Bank, in cyan) and the autophagy regulator Beclin 1 (SantaCruz Biotechnology sc-48381, in red); (**B**) p62 (Progen GP62-C) is labelled in yellow and retinal nuclei are labelled with DAPI (in blue); (**C**) endogenous LC3 staining (Nanotools, 5F10, in magenta) with DAPI nuclear labelling (in blue). Scale bar 20 μm; and (**D**) insets for p62 and LC3 stainings.

In addition to retinal flatmounts, staining can also be performed using eye cryosections or paraffin sections, and immunofluorescence can be used to assess levels of LC3 and other autophagy regulators. Examples of these methods include immunostaining control retinas for Beclin1 and Lamp1 (Figure 4A) and the autophagy substrate p62 (Figure 4B), and endogenous LC3 staining (Figure 4C).

## 7. Flow Cytometry in Retinal Explants

Flow cytometry is a powerful technique that can be used to assess many parameters at the single cell level. This quantitative technique can rapidly analyse large numbers of cells and, when combined with dyes with differential spectral properties, can be used to simultaneously analyse multiple parameters. For example, flow cytometry using selective probes to allow simultaneous assessment of cell viability, levels of free radicals, and/or mitochondrial membrane potential.

Flow cytometry allows quantitative determination of autophagy and autophagic flux in cell lines, with increases in autophagy visualized as decreases in the total GFP signal (see below) [24]. This approach can also be applied to wild-type retinas or retinas isolated from GFP-LC3 or other autophagy-deficient mice, and require prior dissociation of the retinas to the single-cell level by incubation in trypsin.

In embryonic retinas, amino acid starvation results in a decrease in the GFP signal, an effect blocked by simultaneous incubation with protease inhibitors [20]. During autophagy, unbound GFP-LC3 is translocated from the cytoplasm to the autophagosomal membranes and, subsequently, to autophagolysosomes. It should be noted that the acidic milieu of lysosomes quenches GFP fluorescence. Therefore, decreases in GFP fluorescence in a cell can reflect either quenching inside lysosomes or consumption of LC3 during normal autophagosome turnover [25]. This decrease in fluorescence is blocked by lysosomal inhibitors. The same approach can be applied to adult retinas obtained directly from the animal or after explant culture.

## 8. Selective Autophagy: Mitophagy Assays

Although autophagy can degrade bulk parts of the cytoplasm, more and more evidence indicates that is a highly selective process where the substrates to be degraded are selectively targeted for their delivery into the autophagosomes. This applies, for example, for intracellular pathogens, as well as organelles, such as mitochondria, ribosomes, or ER.

**Figure 5:**
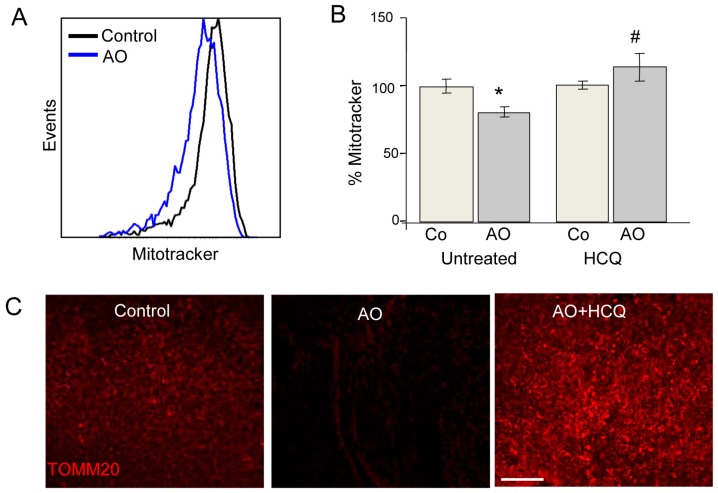
Mitophagy assessment in E13.5 embryonic retinas treated with AO. (**A**) Representative histogram of E13.5 retinas treated for 6 h with AO, dissociated, and stained with MTDR (Invitrogen, M22426) for flow cytometry analysis; (**B**) the percentage mean fluorescence intensity vs. control cells is shown. * *p* < 0.05 with respect to corresponding control group. # *p* < 0.05 with respect to corresponding group incubated in the absence of HCQ or CsA (untreated); and (**C**) TOMM20 staining in retinal flatmounts of E13.5 retinas cultured for 6 h and treated as indicated. Insets show the magnification of TOMM20. Scale bar is 50 μm.

Most studies of mitochondrial autophagy (or mitophagy) are based on the quantification of mitochondria-autophagosome colocalization by fluorescence microscopy or the determination of mitochondrial protein expression by Western blot. We recently developed a new quantitative approach to quantitatively analyse mitophagy. Our flow cytometry-based method assesses mitochondrial mass using Mitotracker Deep Red in combination with several mitophagic and lysosomal inhibitors [26,27]. This method can be applied to both embryonic and adult retinas. In the case of the former, both eyes are required for the assessment of autophagic flux due to the limited amount of tissue available; one retina is incubated in the presence of lysosomal inhibitors while the other serves as a control (Figure 5). Alternatively, in adult retinas, half of the retina can be cultured in vehicle and the other half in the presence of a lysosomal inhibitor, thus reducing animal-to-animal variability. Using this method, we have demonstrated the absolute requirement of mitophagy for neurogenesis in retinal ganglion cells, the first neurons to undergo differentiation in the retina [15]. This method can be easily combined with other fluorescence imaging techniques for the analysis of mitochondrial proteins. For example, TOMM20 and COX IV expression can be evaluated by immunofluorescence and the results compared with protein expression levels measured in the presence of lysosomal inhibitors, thereby allowing assessment of mitophagy flux.

## 9. Conclusions

Autophagy plays an important role in cell homeostasis and the retina constitutes an excellent model in which to assess its function in cells, in general, and in neurons, in particular. Moreover, the fact that retinas can be cultured under semi-natural conditions (organotypic culture), ensuring the preservation of cell-to-cell and cell-to-matrix contacts, provides a more physiological setting than isolated cells. Finally, a wide range of assays can be performed following ex vivo retinal culture, allowing determination of autophagy, mitophagy, and autophagic flux.

## Figures and Tables

**Table 1:** Pharmacological modulators of autophagy. Ref; reference, BRB; blood retinal barrier, BBB, blood brain barrier.

Compound	Source	Dosage	Effect/Mechanism	Used	Permeability
Rapamycin	EuromedexR-5000	100 nM	Autophagy inductor/mTOR kinase inhibitor	Ex vivo	Not tested
AZD8055	Axon, 1561	1 μM	Autophagy inductor/mTOR inhibitor	Ex vivo	Not tested
Bafilomycin A	Sigma, B1793	50 nM	Autophagy inhibitor/vacuolar ATPase inhibitor	Ex vivo	Not tested
3-Methyladenine	Sigma, M9281	10 mM	Autophagy inhibitor/PI3K type III inhibitor	Ex vivo	Not tested
Hydroxichloroquine	Lab.Rubió, 880872.4	30 μg/mL	Autophagy inhibitor/increases lysosomal pH	Ex vivo	Not tested
NH_4_Cl/Leupeptin	Sigma, A9434 Sigma, L2884	20 mM 100 mM	Autophagy inhibitor/increases lysosomal pH-proteases inhibitor	Ex vivo	Not tested
Leupeptin	Sigma, L2884	40 mg/kg	Autophagy inhibitor/proteases inhibitor	In vivo	BRB, not BBB
